# Corporate Social Responsibilities, Psychological Contracts and Employee Turnover Intention of SMEs in China

**DOI:** 10.3389/fpsyg.2021.754183

**Published:** 2021-10-28

**Authors:** Zhang Hui

**Affiliations:** School of Business Administration, Shandong University of Finance and Economics, Jinan, China

**Keywords:** SMEs, corporate social responsibility, transactional psychological contract, relational psychological contract, employee turnover intention

## Abstract

As an important organizational strategy and action that affects employee perception and attitude, corporate social responsibility is essential for small and medium-sized enterprises (SMEs) to reduce turnover rate and achieve sustainable growth. This paper integrates social identity theory and social exchange theory to construct an external reputation mechanism and internal trust mechanism to explore the influence mechanism of corporate social responsibility on employee turnover intention and the intermediate transmission mechanism of psychological contract. The research results show that corporate social responsibility has a significant negative impact on employee turnover intention. Compared with external corporate social responsibility, internal corporate social responsibility has a stronger negative impact on employee turnover intention; corporate social responsibility has a significant negative impact on employee transactional psychological contract, while corporate social responsibility has a significant positive impact on employee relational psychological contracts; transactional psychological contract has a significant positive effect on employee turnover intention, while relational psychological contract has a significant negative effect on employee turnover intention; psychological contract has a significant and complete mediating effect on the relationship between external corporate social responsibility and employee turnover intention, and the psychological contract plays a significant part of the intermediary role between the internal corporate social responsibility and the employee turnover intention. The conclusions enriches the complex relationship between corporate social responsibility and employee turnover intentions, and provides a reference for SMEs to effectively perform internal and external social responsibilities and reduce employee turnover rates.

## Introduction

As an important part of the socialist market economy, SMEs are an important force to promote the economic and social development of China (Du et al., 2020). However, the 2020 new crown pneumonia epidemic has brought unprecedented challenges to Chinese social and economic development. More than 30 million SMEs are unable to give full play to the economies of scale and resource advantages due to their small scale, weak anti-risk capabilities, and scarce resources (Aikaeli, 2012). Due to the unfavorable external environment, they are on the verge of bankruptcy at any time, and the phenomenon of employee turnover is becoming more and more serious. Frequent employee departures will not only affect the sustainability of corporate team culture and knowledge capital development, but also increase the company's labor conversion costs to a certain extent, weaken organizational commitment, and cause other employees to form negative psychological perceptions of the company (Wang et al., 2017). Corporate social responsibility is crucial for organizations that try to influence employees' attitudes to pursue sustainable development and competitive advantage (Chen and Donna, 2020) as an important organizational strategy and action that affects employees' perceptions and attitudes (Ma et al., 2018). The fulfillment of social responsibilities by SMEs is conducive to maintaining long-term relationships with stakeholders, gaining a good social reputation, enhancing core competitive advantages, and alleviating the problem of employee turnover. Therefore, corporate social responsibility is regarded as an important research topic in the field of management, and it has also become the mainstream practice of organizations (Faisal et al., 2020).

However, SMEs in China are confronted with some issues, such as environmental pollution, over-utilization of resources and lack of social responsibilities such as employment relations problems driven by economic interests in recent years, which have affected the sustainable growth of enterprises and the harmonious development of society to a certain extent. In terms of external stakeholders, some scholars have explored the influence mechanism of corporate social responsibility on corporate financial performance (Waddock and Graves, 1997), consumers (Luo and Bhattacharya, 2006) and corporate reputation (Johnson, 2003). Although some scholars have initially explored the positive correlation between corporate social responsibility and employee turnover rate based on the social identity theory (Ng et al., 2019), they have not explored the impact path of corporate social responsibility on employee turnover intention based on the level of employees as an internal interest group (Chen and Donna, 2020; Fang et al., 2021). This led to the research on the underlying mechanism of employee perception of corporate social responsibility is still “fragmented and incomplete” (Gond et al., 2017). As the company's core resource and important stakeholder group (Nie, 2015), employees are an important force in the creation of heterogeneous resources and value creation of the company's core competitiveness (Huang and Wang, 2016). The social exchange theory proposes that employees believe that they are obligated to repay the care and support given by the organization (Eisenberger et al., 1986), so employees' perception of corporate social responsibility will affect their behavioral decisions (Rousseau, 1989).

Psychological contract is the employees' cognition of the responsibilities of both employers and employees (Rousseau, 1989) and an important manifestation of the quality of exchange relationship between employees and organizations (Wang et al., 2003). Employees' perception of corporate social responsibility will affect their judgments on the importance and value of their roles in their work, thereby weakening or enhancing the positive effects of the work on themselves (Liu and Zhou, 2017). To sum up, although the existing research results are based on different theoretical perspectives and explore the relationship between corporate social responsibility and employee turnover rate, however, they have not analyzed in depth the differentiation paths of different types of corporate social responsibilities that affect employee turnover intentions. Therefore, it is not conducive to systematically and comprehensively clarify the influence mechanism of corporate social responsibility on employee turnover intention. In view of this, this paper focuses on SMEs in China and explores the complex relationship between internal and external corporate social responsibilities and employee turnover intention. By constructing an external social reputation mechanism and internal trust mechanism of enterprises, this paper clarifies the differentiated intermediate conduction impact of transactional psychological contracts and relational psychological contracts in the impact of corporate social responsibility on employee turnover intention in order to provide a theoretical reference for SMEs to better fulfill their internal and external social responsibilities, and thereby reduce the employee turnover intention.

## Theoretical Analysis and Hypothesis

### Corporate Social Responsibility and Employee Turnover Intention

Corporate social responsibility refers to the organizational behavior of enterprises that pay attention to the needs and expectations of stakeholders in strategic decisions and actions, voluntarily allocate limited resources, and achieve sustainable social, economic and environmental development (Aguinis and Glavas, 2019). The stakeholders of corporate social responsibility mainly include external government and other regulatory agencies, social media, suppliers, customers, consumers and community residents and internal corporate employees (Behroozz et al., 2018). The fulfillment of corporate social responsibility is conducive to the establishment of a good social image for the company, which in turn improves the organizational recognition of employees. The employee turnover intention refers to the employee's subjective estimation of the possibility of leaving the company (Mowday et al., 1982), and the behavior preference that ultimately leads to his leaving the company (Mobley et al., 1979). In addition, related studies have shown that employees' perception of corporate social responsibility can more effectively and directly affect their attitudes and behaviors (Yan et al., 2020), so this paper focuses on the influence mechanism of corporate social responsibility perception on employee turnover intention.

### External Reputation Mechanism

The social identity theory believes that consciously fulfilling social responsibilities can effectively enhance employees' sense of belonging and unity to the organization. Employees are more able to recognize the image of a company with a sense of social responsibility on the basis of being aware of the value of organizational membership. The higher the employees' sense of identity with the organization is, it is more conducive to maintaining a lasting employment relationship between employees and the organization, meeting respected social needs, strengthening employees' pride, thereby reducing their turnover intention and inhibiting the emergence of deviant behaviors in the workplace (Faisal et al., 2020).

On the one hand, companies donate products or cash to the society through the cause-related marketing activities, which is conducive to the establishment of a good image in the eyes of the public and a higher social reputation, which can meet the self-respect needs of employees to a certain extent. The more employees' needs are met, the more they can enhance their sense of identity and attachment to the organization (Zhang et al., 2014), encourage employees to adopt positive work attitudes and behaviors and reduce their turnover intention (Dutton, 1994). On the other hand, companies can improve resource use efficiency, reduce environmental pollution levels and reduce the harm to biodiversity by building an environmental management system and strictly implementing environmental protection policies such as energy saving and emission reduction. The series of activities carried out by enterprises in response to the external environment are conducive to consumers forming a positive evaluation of the performance of corporate social responsibility. While affirming the company's green behavior, it also generates more off-role behaviors, such as proactively promoting the corporate image to others, recommending corporate products, etc. (Elif et al., 2016), so as to gain a good social reputation among the consumers. In addition, companies strictly abide by quality and safety standards to ensure product quality and safety, meet the diverse needs of different consumers for products, and provide consumers with more transfer value, which is conducive to the recognition and appreciation of consumers and government regulatory agencies. When employees perceive that the more the company cares about the interests of the consumers, the more they can affirm their self-worth, and then have a sense of identity with the company and reduce their turnover intention (Zhao and Tong, 2018).

It can be seen that, companies are conducive to building a good social reputation mechanism by fulfilling social responsibilities to external stakeholders, thereby enhancing employees' sense of pride and dependence on the organization and reducing employee turnover intention (Liu, 2015). In view of this, this paper proposes hypothesis H1:

H1: External corporate social responsibility has a significant negative impact on employee turnover intention

### Internal Trust Mechanism

Social exchange theory believes that when organizations provide employees with good promotion space, benefits and labor remuneration, employees will intend to invest more trust and loyalty to maintain their contractual relationship with the organization, generate a sense of responsibility for reward, and voluntarily adopt extra-role behaviors that are beneficial to the organization (Yan et al., 2020).

First of all, the more a company provides employees with job training opportunities and makes them feel that they have an ideal promotion space in the company, the stronger their trust in the company is, and the more they can consciously perform their job responsibilities and reduce their turnover intention (Wang et al., 2017). Secondly, the more the compensation system formulated by the company can meet the needs of employees, the more conducive it is to promote employees' loyalty to the organization and create more value (Onyishi et al., 2020). Therefore, the less likely it is to leave the company. In addition, the more a company creates a good working environment and an internal fairness atmosphere for its employees, the more conducive it is to improve the cooperative relationship between the company and its employees, enhance organizational cohesion, increase employees' job satisfaction and organizational commitment, and reduce their turnover intention (Li et al., 2012).

In summary, companies can help form a good internal trust mechanism, increase employees' satisfaction and loyalty to the company, and reduce their turnover intention by fulfilling social responsibilities about employees. Therefore, this paper proposes hypothesis H2:

H2: Internal corporate social responsibility has a significant negative impact on employees turnover intention

### Corporate Social Responsibility and Psychological Contract

Psychological contract is not only an employees' cognition of the responsibilities of both employers and employees (Rousseau, 1989), but also an important manifestation of the quality of the exchange relationship between employees and organizations (Wang et al., 2003), and it plays a role as a communication bridge between the company and employees. When employees perceive that the company has fulfilled their social responsibilities such as promotion, high remuneration, performance rewards and career development, employees often choose to work harder, keep loyal to the company, pre-notify before leaving and other organizational citizenship behaviors. Psychological contract includes two aspects, such as transactional psychological contract (TPC) and relational psychological contract (RPC), (Raja et al., 2004). Among them, the transactional psychological contract means that employees regard work as a means of earning a living, and are more concerned about the immediate material rewards and short-term employment relationships (Liu and Zhou, 2017); while the relational psychological contract focuses on the long-term and stable relationship and development between employees and the organization in the future (Raja et al., 2004).

On the one hand, when the company fails to fulfill the corresponding social responsibilities toward external stakeholders, that is, the company fails to provide consumers with satisfactory products or services, fails to provide a good ecological environment for community residents (Tian et al., 2021), or fails to take the initiative to provide the public disclosure of relevant financial statements, etc. (Sabrina et al., 2019), employees perceive the low level of corporate social responsibility. The company has a poor reputation and social image in the network of external groups, leading the employees to doubts about the value and importance of its role in work, which weakens the positive effect of work on employees to a certain extent (Liu and Zhou, 2017). Based on this, employees regard work as merely a means of earning a living, and believe that their responsibilities to the company are limited to general job responsibilities which leading employees to pay more attention to short-term material returns and employment relationships, and tend to establish a transactional psychology contract with the company (Liu and Zhou, 2017). Similarly, when the company fails to fulfill the corresponding social responsibilities for internal stakeholders, fails to provide employees with generous benefits or promotion opportunities, or fails to develop career plans for employees, etc. (Sabrina et al., 2019), employees perceive the company failure to fulfill social responsibilities well. It is difficult for companies to gain a sense of identity within the network of internal employee groups (Elakremi et al., 2018), resulting in employees not believing that the company can truly consider employees' development and achieve sustainable growth. Therefore, employees pay more attention to the short-term material benefits given by the company, separate their personal development goals from the company's strategic goals, and form a transactional psychological contract (Kang and Sung, 2019).

On the other hand, when the company treats external stakeholders fairly and provides them with a large amount of products or cash donations, they consciously adopt green behaviors to save resources and protect the environment (Sabrina et al., 2019), or fully consider the interests of internal employees, and provide them with a large number of promotion opportunities or with generous benefits, employees perceive a higher level of corporate social responsibility, which is conducive to enhancing employees' sense of trust and identification with the company (Evans and Davis, 2014), which in turn promotes the formation of high-quality social exchange relationships between employees and the company. Based on this, employees pay more attention to their own long-term development goals, try to integrate them with the company's strategic goals and tend to establish a relational psychological contract with the company (Liu and Zhou, 2017).

It can be seen that employees tend to form relational psychological contract when they perceive a high level of corporate social responsibility to stakeholders; and when they perceive a low level of corporate social responsibility to stakeholders, employees are more tend to form a transactional psychological contract. Therefore, this paper proposes the following hypotheses:

H3: External corporate social responsibility has a significant negative impact on transactional psychological contract
H4: External corporate social responsibility has a significant positive impact on relational psychological contract
H5: Internal corporate social responsibility has a significant negative impact on transactional psychological contract
H6: Internal corporate social responsibility has a significant positive impact on relational psychological contract

### Psychological Contract and Employee Turnover Intention

Social exchange theory believes that when an employee forms the employment relationship with an enterprise, he signs a reciprocal normative psychological contract with the enterprise (Rousseau, 1998). The psychological contract clearly stipulates the fair treatment that the company should provide to employees, so as to encourage employees to produce expectations that organizations pay enough attention to them, care about their happiness (Griffeth et al., 2000), and provide them with continuous fair treatment (Moorman et al., 1998). However, when they perceive that the company has violated the psychological contract, employees will have emotional exhaustion (Johnson and O'Leary-Kelly, 2003), which may generate a higher willingness to quit and other reactions or behaviors (Zhao et al., 2007; Kang and Sung, 2019).

Employee transactional psychological contract pay more attention to the short-term economic exchanges between them and the company. They often regard work as a means of earning a living. They like to strictly stipulate working hours and only work within the time period stipulated in the contract, and it is difficult for them to agree with the company's strategic goals instead of pursuing their own short-term goals (Raja et al., 2004). Once the high-level transactional psychological contract is broken or violated, employees' pride in their work gradually decreases, and they no longer invest time to maintain the consistency between cognition and behavior (Liu and Zhou, 2017), so employees are more likely to resign Willingness, and then choose to leave the enterprise. However, the employee relational psychological contract emphasizes the long-term emotional exchange with the enterprise, regards itself as a member of the enterprise team, and expects continuous growth within the enterprise. In addition, employees who hold a relational psychological contract believe that by working hard, they can obtain reasonable promotion opportunities and fair rewards, benefits or remuneration (Raja et al., 2004). Therefore, once the high-level relational psychological contract is destroyed or violated, employees will not immediately have the intention to leave and end their employment relationship with the company (Behroozz et al., 2018). Based on this, this paper proposes hypotheses H7 and H8:

H7: Transactional psychological contract has a significant positive impact on employee turnover intention
H8: Relational psychological contract has a significant negative impact on employee turnover intention

### The Intermediary Influence of Psychological Contract

The interaction between people is essentially an exchange relationship, such as material exchange and the exchange of non-material resources such as emotion and reputation (Blau, 1964). The relationship between employees and the organization is manifested as a mutual dependence based on the exchange of resources, and its essence is an exchange relationship. Rousseau (1998) further divides the psychological contract into two types including transactional psychological contract and relational psychological contract. Psychological contract is an employees' understanding or belief in the responsibilities and obligations between the employers and employees. And this understanding or belief is manifested by employees' commitment and perception of the exchange relationship between their external and internal contributions (effort, ability, loyalty, etc.) and corporate incentives (remuneration, promotion, job security, etc.). Among them, the transactional psychological contract is suitable for the exchange of economic resources and has the characteristics of short-term economic exchange, while the relational psychological contract pays more attention to the long-term social emotional exchange relationship in addition to economic exchange, therefore, it is suitable for the exchange of social emotional resources (Raja et al., 2004).

In addition, psychological contract is particularly important for employees' attitudes and behaviors as an employees' cognition of employment responsibilities. The social cognition theory believes that the transformation of stimulus conditions into corresponding follow-up behaviors must rely on the mediation of a series of cognitive processes (Wang and Wang, 1992). As an important stimulus, corporate social responsibility affects employees' attitudes or behaviors through the intermediary mechanism of psychological contracts. When they perceive that the company fulfills a lower level of social responsibility, employees tend to form a transactional psychological contract, and employees with short-term, specific responsibility perception experience regard work as a means of earning a living (Liu and Zhou, 2017). Therefore, they choose as few organizational commitments or civic behaviors as possible to maintain the consistency between cognition and behavior (Liu and Zhou, 2017). The degree of commitment and pride to the work is lower, and the willingness to leave is more likely to occur. The lower the level of a company's external social responsibility is, the lower the evaluation of the company's social responsibility activities by the outside world is. To a certain extent, the low social reputation makes employees less likely to have a sense of identity with the company (Elakremi et al., 2018), thus tending to form a transactional psychology contract and give rise to the idea of leaving the enterprise. In the same way, the lower the level of corporate social responsibility is, the more difficult it is for employees to obtain reasonable training and promotion opportunities, generous benefits and salaries, etc., and the lower the level of satisfaction and trust in the company is, and the more likely it is to form transactional psychological contract, which makes employees more likely to generate turnover intention (Kang and Sung, 2019).

On the contrary, when they perceive that the company fulfills a higher social responsibility, employees show a sense of responsibility for the long-term development of relationships at work, so it is easier to establish a relational psychological contract. This sense of responsibility can stimulate employees to reflect on their moral responsibilities and internalize their moral values, thereby voluntarily generating more organizational commitments or civic behaviors, enhancing their sense of identity and belonging to the company, and reducing their willingness to leave. The higher the level of a company's external social responsibility is, the easier it is for employees to obtain positive evaluation information on corporate social responsibility activities through oral communication or other media (Smidts and Erasmus, 2001). This type of information can enhance employees' positive evaluation of corporate reputation and sense of social identity (Elakremi et al., 2018), enhance employees' self-worth and satisfy their psychological needs such as a sense of belonging (DeRoeck et al., 2014), thereby forming a relational psychological contract. On this basis, employees are more willing to work for companies with a sense of social responsibility (Rupp and Mallory, 2015), actively consider the long-term interests of the company, adopt more attitudes or behaviors that are conducive to the development of the company, and reduce their turnover intention (Behroozz et al., 2018). In the same way, the higher the level of the company's fulfillment of internal social responsibilities is employees can get more job promotion opportunities and generous benefits. Based on the reciprocal norms, employees will be more actively involved in their job roles and pay more energy, emotions and material resources as the company's return or response to employees for fulfilling their social responsibilities (Cropanzano and Mitchell, 2005). In addition, the higher the level of internal social responsibilities is, it is more conducive to improve employees' satisfaction, loyalty, and sense of trust with the company (Peterson, 2004), thereby building a relational psychological contract. Based on this, employees are more willing to pay attention to the long-term cooperative relationship with the enterprise, and even sacrifice their short-term interests to fulfill the long-term interests of the enterprise and contribute to the overall performance of the enterprise (Zhao et al., 2019), so the less likely it is to generate a willingness to leave (Chaudhary, 2017).

To sum up, the influence of corporate social responsibility on employee turnover intention is realized through an intermediary mechanism, that is, internal and external corporate social responsibility acts on transactional psychological contract and relational psychological contract, thereby affecting employee turnover intention. In view of this, this paper proposes hypothesis H9:

H9: Psychological contract plays a significant intermediary role in the negative impact of corporate social responsibility on employee turnover intention
H9a: Transactional psychological contract plays a significant intermediary role in the negative impact of external corporate social responsibility on employee turnover intention
H9b: Transactional psychological contract plays a significant intermediary role in the negative impact of internal corporate social responsibility on employee turnover intention
H9c: Relational psychological contract plays a significant intermediary role in the negative impact of external corporate social responsibility on employee turnover intention
H9d: Relational psychological contract plays a significant intermediary role in the negative impact of internal corporate social responsibility on employee turnover intention

**Figure 1:**
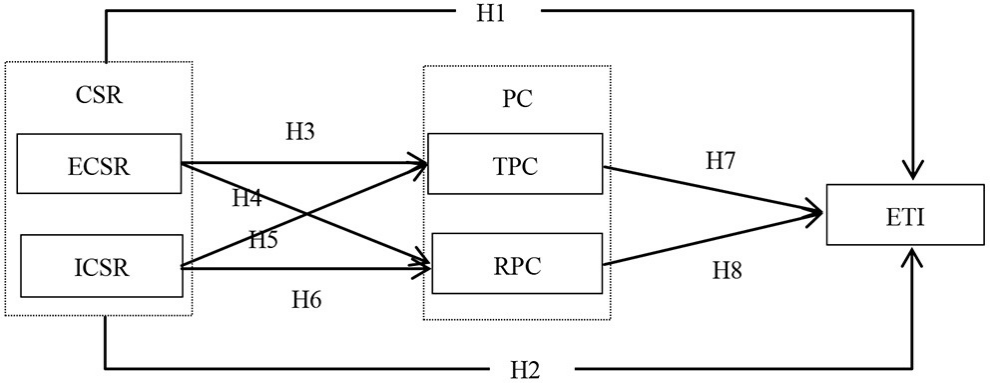
Theoretical model diagram.

The theoretical model diagram of this paper is shown in Figure 1.

## Research Design

### Variable Measurement

The measurement scale used in this paper is a mature scale that has been verified at home and abroad. In order to ensure that the foreign language scale can be accurately understood, the researchers used a translation-back translation program to process the scale accordingly. The measurement scales of this paper all use Likert 5-level scoring method, “one” means completely disagree, and “five” means completely agree.

#### Corporate Social Responsibility

Drawing on the scale developed by Sabrina et al. (2019), internal corporate social responsibility (ICSR) includes “your company supports the professional development of employees,” “your company claims to provide employees with generous maternity leave welfare,” “your company claims to provide employees with flexible working hours” and other six items. External corporate social responsibility (ECSR) is mainly measured from four levels such as corporate donations, emission reduction, citizenship and product safety, including “your company will donate products to the society,” “your company has issued policies on energy conservation and emission reduction,” “your company encourages employees to actively participate in voluntary activities,” “your company has taken relevant measures to ensure that the product label is clear and effective” and other 11 items.

#### Psychological Contract

Learning from Raja et al. (2004) about the maturity scale of psychological contract, the psychological contract is divided into two dimensions such as transactional contract and relational contract. Among them, the transactional contract includes nine topics such as “I am loyal to the company because of contract regulations,” “I prefer to strictly regulate working hours,” and “I only do what is necessary to complete the work.” The relational contract also covers “I look forward to continuous growth in the company,” “If I work hard, there will be a reasonable opportunity for promotion,” “I think the company will give corresponding rewards to employees for their efforts and dedication,” etc.

#### Employee Turnover Intention

Drawing lessons from Kang and Sung (2019) on the maturity scale of employee turnover intentions, this paper uses three questions such as “I have a lot of ideas about leaving the company,” “I am actively looking for new employment opportunities” and “As long as possible, I will leave this company” to characterize the employee turnover intention.

#### Control Variables

Learning from Wang et al. (2017) and Behroozz et al. (2018) related maturity scales, the employee's gender (Gender), age (Age), education level (Education) and working years (Year) are set as the control variables of this paper.

### Reliability and Validity Test

As shown in Table 1, the Cronbach's α values of each variable measurement scale in this paper are all >0.7, indicating that each variable measurement scale has good reliability. The KMO value of each variable measurement scale is >0.7, and the minimum value of the Bartlett sphere chi-square test is 629.330 and it reaches the significance level (*p* = 0.000 < 0.001) indicating that each scale passed the validity test.

**Table 1:** Reliability and validity test results of each variable.

**Variable name**	**Dimension**	**Items number**	**Cronbach's α**	**KMO**	**Bartlett sphere chi-square**	**Cumulative proportion of total variance**
CSR	ICSR	6	0.967	0.899	1,599.550^***^	85.808%
	ECSR	11	0.985	0.919	4,286.269^***^	86.934%
PC	TPC	9	0.919	0.872	1,331.540^***^	61.109%
	RPC	9	0.990	0.941	4,015.496^***^	92.658%
ETI	ETI	3	0.950	0.769	629.330^***^	91.026%

*** *p < 0.001*.

## Empirical Analysis

### Descriptive Statistical Analysis

During the period from June 2021 to August 2021, this paper used effective means such as on-site interviews and e-mails to distribute a total of 286 questionnaires relying on SMEs in Shandong, Zhejiang, Hebei and Jiangsu provinces, and 209 valid questionnaires were returned. There are a large number of SMEs in China and their geographical distribution is wide. This paper selects SMEs in Shandong, Zhejiang, Hebei, and Jiangsu Provinces as the sample companies because the number of SMEs in the above four provinces accounts for about 30% of the national total, which is highly representative. In addition, the above-mentioned four provinces are located in the eastern coastal areas of China, with relatively developed economies and high level of fulfillment of social responsibilities by SMEs, so they are suitable as research objects. The effective response rate was 73.08%. The surveyed companies covered the service industry, manufacturing industry, finance and insurance industry, wholesale and retail industry and other industries. The characteristics of each sample are as follows: (1) Gender. Men accounted for 29.7% and women accounted for 70.3%. (2) Age. The survey respondents between the ages of 19–25 accounted for 51.2%, the survey respondents between the ages of 26–30 accounted for 5.3%, the survey respondents between the ages of 31–35 accounted for 8.6%, those between the ages of 36–40 accounted for 10.5%, those aged 41–45 accounted for 12.4%, those aged 46–50 accounted for 8.6%, and those aged 51–55 accounted for 3.3%. (3) Education level. Primary school education accounted for about 0.4%, junior high school education accounted for 1.4%, technical secondary school or high school education accounted for 8.1%, university education accounted for about 84.6%, master degree accounted for about 3.8%, doctor degree accounted for about 1.4%. (4) Working years. 3 years or less working years accounted for 63.1%, 4–6 years accounted for 10.0%, 7–10 years accounted for 11.0%, 11–15 years accounted for about 8.6%, 16–20 years accounted for about 3.8%, 21–25 years accounted for about 2.3%, 26–30 years accounted for about 0.4%, more than 30 years accounted for about 0.4%. It can be seen that the age, education level and working years of the survey subjects all show a large span, and thus they have a strong representativeness.

### Correlation Analysis

As shown in Table 2, there is no significant relationship between employee age, working years and turnover intention, while there is a significant negative correlation between employee gender and turnover intention, that is, compared with male employees, female employees have lower willingness to leave. There is a significant positive correlation between employees' education level and turnover intention, that is, with the improvement of their educational level, they have more opportunities for employment choices, and therefore their willingness to leave is stronger. In addition, there is a significant positive correlation between internal and external corporate social responsibilities, and both have a significant negative relationship with turnover intention, a significant negative relationship with transactional psychological contract, and a significant positive relationship with relational psychological contract. There is a significant positive correlation between transactional psychological contract and employee turnover intention, while there is a significant negative relationship between relational psychological contract and employee turnover intention.

**Table 2:** Correlation analysis results.

**Variables**	**1**	**2**	**3**	**4**	**5**	**6**	**7**	**8**	**9**
1 ICSR	1								
2 ECSR	0.609^***^	1							
3 TPC	−0.552^***^	−0.679^***^	1						
4 RPC	0.533^***^	0.486^***^	−0.442^***^	1					
5 ETI	−0.410^***^	−0.266^***^	0.378^***^	−0.445^***^	1				
6 Gender	0.170^*^	0.109	−0.155^*^	0.116	−0.161^*^	1			
7 Age	−0.122	−0.109	0.060	−0.005	0.027	−0.045	1		
8 Education	−0.058	−0.066	0.007	−0.295^***^	0.147^*^	−0.071	−0.102	1	
9 Year	−0.148^*^	−0.095	0.046	0.043	−0.020	−0.118	0.839^***^	−0.202^**^	1
Average	3.161	3.141	3.530	3.246	3.321	1.703	2.670	3.942	1.914
SD	1.169	1.145	0.907	1.153	0.989	0.458	1.981	0.525	1.452

*N = 209;*

*** *p < 0.001*,

** *p < 0.01*,

* *p < 0.05; two-tailed test*.

### Regression Analysis

#### Main Effect Test

As shown in Table 3, model M1 adds the independent variable external corporate social responsibility on the basis of M0 to test the effect of external corporate social responsibility on employee turnover intention. Regression analysis results show that external corporate social responsibility has a significant negative impact on employee turnover intention (β = −0.244, *p* < 0.001), indicating that the more employees perceive the company's performance of external social responsibilities, the more they can reduce their willingness to leave. As a result, H1 passed the inspection. In the same way, model M2 adds independent variable internal corporate social responsibility on the basis of M0 to test the effect of internal corporate social responsibility on employee turnover intention. Regression analysis results show that internal corporate social responsibility also has a significant negative impact on employee turnover intention (β = −0.398, *p* < 0.001), that is, the more employees perceive the company's fulfillment of internal social responsibilities, the more they can restrain their ideas of leaving the company and reduce their turnover intention, so H2 passes the test. In addition, compared with external corporate social responsibility, internal corporate social responsibility has a stronger negative impact on employee turnover intention.

**Table 3:** The impact of corporate social responsibility on employee turnover intention.

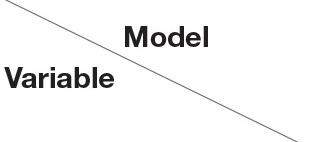	**ETI**								
	**M0**	**M1**	**M2**	**M3**	**M4**	**M5**	**M6**	**M7**	**M8**
**Control variables**									
Gender	−0.162^*^	−0.138^*^	−0.103	−0.105	−0.119	−0.105	−0.116	−0.087	−0.096
Age	0.150	0.125	0.149	0.113	0.110	0.113	0.107	0.128	0.122
Education	0.122	0.104	0.091	0.124	0.002	0.124	0.006	0.101	0.021
Year	−0.140	−0.143	−0.197	−0.119	−0.108	−0.119	−0.111	−0.169	−0.153
**Independent variables**									
ECSR		−0.244^***^				−0.003	−0.060		
ICSR			−0.398^***^					−0.283^***^	−0.245^**^
**Mediating variables**									
TPC				0.360^***^		0.358^***^		0.208^**^	
RPC					−0.426^***^		−0.395^***^		−0.290^***^
R^2^	0.032	0.087	0.181	0.156	0.195	0.152	0.193	0.207	0.232
ΔR^2^	0	0.055	0.149	0.124	0.163	0.065	0.106	0.026	0.051
*F*	2.723^*^	4.952^***^	10.198^***^	8.707^***^	11.049^***^	7.220^***^	9.309^***^	10.073^***^	11.485^***^

*N = 209;*

*** *p < 0.001*,

** *p < 0.01*,

* *p < 0.05*.

#### Mediating Effect Test

As shown in Table 4, model M1 adds the independent variable external corporate social responsibility on the basis of M0 to test the impact of external corporate social responsibility on employee transactional psychological contracts. Regression analysis results show that external corporate social responsibility has a significant negative impact on employee transactional psychological contracts (β = −0.675, *p* < 0.001), that is, the more employees perceive the company's performance of external social responsibilities, the more likely it is to form transactional psychological contracts, so H3 passes the test. Similarly, model M2 adds independent variable internal corporate social responsibility on the basis of M0 to test the effect of internal corporate social responsibility on employee transactional psychological contracts. Regression analysis results show that internal corporate social responsibility also has a significant negative impact on employee transactional psychological contracts (β = −0.550, *p* < 0.001). Therefore, hypothesis H5 can be verified. Both external corporate social responsibility (β = 0.466, *p* < 0.001) and internal corporate social responsibility (β = 0.526, *p* < 0.001) both have a significant positive impact on employee relational psychological contracts. It is assumed that H4 and H6 pass the test respectively.

**Table 4:** The impact of corporate social responsibility on psychological contract.

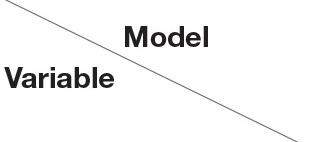	**TPC**			**RPC**		
	**M0**	**M1**	**M2**	**M3**	**M4**	**M5**
**Control variables**						
Gender	−0.157^*^	−0.091	−0.076	0.101	0.056	0.023
Age	0.102	0.033	0.101	−0.093	−0.045	−0.092
Education	−0.006	−0.054	−0.048	−0.282^***^	−0.249^***^	−0.242^***^
Year	−0.059	−0.067	−0.138	0.076	0.081	0.152
**Independent variables**						
ECSR		−0.675^***^			0.466^***^	
ICSR			−0.550^***^			0.526^***^
R^2^	0.009	0.458	0.298	0.081	0.293	0.346
ΔR^2^	0	0.449	0.289		0.212	0.265
*F*	1.454	36.111^***^	18.674^***^	5.593^***^	18.236^***^	22.983^***^

*N = 209;*

*** *p < 0.001*,

* *p < 0.05*.

In addition, as shown in Table 3, model M3 adds an intermediary variable transactional psychological contract on the basis of M0 to test the effect of transactional psychological contract on employee turnover intention. Regression analysis results show that transactional psychological contract has a significant positive impact on employee turnover intention (β = 0.360, *p* < 0.001), that is, the more an employee forms a transactional psychological contract, the more likely it is to have the idea of leaving. Therefore, H7 is verified. In the same way, model M4 adds an intermediary variable relational psychological contract on the basis of M0 to test the effect of relational psychological contract on employee turnover intention. Regression analysis results show that the relational psychological contract has a significant negative impact on employee turnover intention (β = −0.426, *p* < 0.001), that is, the more an employee forms a relational psychological contract, the less likely it is to have the idea of leaving, so H8 passes the test. Model M5 adds an intermediary variable transactional psychological contract on the basis of M1 to test the intermediary role of transactional psychological contract in the relationship between external corporate social responsibility and employee turnover intention. The regression analysis results show that transactional psychological contract has a complete mediating effect between the external corporate social responsibility and the employee turnover intention, so the hypothesis H9a passes the test. That is, the external corporate social responsibility completely affects the employee turnover intention through the transactional psychological contract. Similarly, model M6 shows that the relational psychological contract also has a complete mediating effect between the external corporate social responsibility and the employee turnover intention, so the hypothesis H9c passes the test. Model M7 shows that the transactional psychological contract has a partial mediating role in the negative influence of internal corporate social responsibility on the employee turnover intention, so the hypothesis H9b passes the test. Model M8 shows that relational psychological contract also has a partial mediating role in the negative impact of internal corporate social responsibility on employee turnover intention, so the hypothesis H9d passes the test. To sum up, the psychological contract plays a significant mediating role in the negative impact of corporate social responsibility on employee turnover intention. Therefore, hypothesis H9 passes the test.

## Conclusion and Enlightenment

### Conclusion

(1) Corporate social responsibility has a significant negative impact on employee turnover intention. On the one hand, external corporate social responsibility has a significant negative impact on employee turnover intention. The fulfillment of external social responsibilities by SMEs is conducive to the formation of a good reputation and positive evaluation from the public, thereby enhancing employees' sense of identity with the company and reducing their willingness to leave. On the other hand, internal corporate social responsibility also has a significant negative impact on employee turnover intention. The fulfillment of internal social responsibilities by SMEs is conducive to enhancing employees' sense of trust and belonging to the enterprise, and reducing their willingness to leave. In addition, compared with external corporate social responsibility, internal corporate social responsibility has a stronger negative impact on employee turnover intention. The reason may lie in the more direct perception of internal social responsibilities from employees.

(2) Corporate social responsibility has a different impact on employee psychological contract. Corporate social responsibility has a significant negative impact on employee transactional psychological contract. The higher the level of internal and external social responsibility performed by an enterprise is, the easier it is for employees to have a stronger sense of identity and trust with the enterprise. Therefore, as a response and feedback for the enterprise to fulfill its social responsibilities, employees are more inclined to form a relational psychology contract, not transactional psychological contract.

(3) Psychological contract has a different effect on employee turnover intention. On the one hand, transactional psychological contract has a significant positive impact on employee turnover intention. The employees who form the transactional psychological contract pay more attention to short-term material benefits and believe that work is only a means of earning a living, so their devotion and enthusiasm for work is low. Once the transactional psychological contract is destroyed, employees will often immediately have the idea of leaving and choose to end their employment relationship with the company. On the other hand, the relational psychological contract has a significant negative impact on employee turnover intention. Employees who form a relational psychological contract pay more attention to long-term emotional relationships and believe that they have a strong sense of belonging and value as a member of the company, so they have a higher level of satisfaction and commitment to their work, and they will not easily generate the idea of leaving the company.

(4) Psychological contract has a significant mediating role in the relationship between corporate social responsibility and employee turnover intention. On the one hand, the psychological contract has a significant and complete mediating effect between the external corporate social responsibility and the employee turnover intention. Psychological contract plays a completely intermediary role in the negative influence of external corporate social responsibility on employee turnover intention, that is, the negative influence of corporate social responsibility on employee turnover intention is completely realized through the intermediary transmission effect of psychological contract. The reason may be that the role of external corporate social responsibility mainly relies on the reputation mechanism. Employees use social media reports or public publicity to indirectly perceive the performance of corporate external social responsibilities, and it is difficult to determine whether to stay in the company through direct feelings. Therefore, the influence of external corporate social responsibility on employee turnover intention needs to be realized with the effect of psychological contract. On the other hand, the psychological contract plays a significant part mediating role between the internal corporate social responsibility and the employee turnover intention. In comparison, internal corporate social responsibility can directly affect employee turnover intention through the trust mechanism, so the psychological contract only plays a part of the intermediary role in this influencing process.

### Theoretical Contributions

This paper integrates social identity theory and social exchange theory to construct an external reputation mechanism and internal trust mechanism to explore the influence mechanism of corporate social responsibility on employee turnover intention and the intermediate transmission mechanism of psychological contract. The conclusions expand the application boundaries of social identity theory and social exchange theory, subdivide corporate social responsibility into internal and external levels, which can enrich the research scope of corporate social responsibility, and clarify the driving factors of employee turnover intention and their different influence mechanisms, which provides a theoretical reference for follow-up related research.

### Management Enlightenment

(1) SMEs should take into account internal and external social responsibilities. In view of the fact that corporate social responsibility can effectively reduce employees' willingness to leave through external reputation mechanism and internal trust mechanism, companies should take into account both internal and external social responsibilities. On the one hand, SMEs must fully fulfill their external social responsibilities, such as providing consumers with satisfactory products or services, and providing community residents with a good ecological environment, so as to form a good reputation and image within the network of external groups and reduce employee turnover intention. On the other hand, considering that internal corporate social responsibility has a stronger negative impact on employee turnover intention, and employees can more directly and effectively perceive the internal corporate social responsibilities, SMEs should pay more attention to internal employees and consciously perform social responsibility in order to provide employees with more job promotion opportunities and better benefits, enhance employees' sense of identity and trust with the company, and reduce their willingness to leave.

(2) SMEs should actively guide employees to establish relational psychological contracts. Considering that the relational psychological contract negatively affects employee turnover intention, SMEs should actively guide employees to establish relational psychological contract. While fulfilling their internal social responsibilities, SMEs should strengthen communication and exchanges between the company and employees, pay attention to and meet the needs of employees, so as to guide employees to form relational psychological contract. On this basis, employees will invest more time and energy in their work, combine corporate strategic goals with their own long-term development goals, and strive to maintain their emotional and social exchange relationships with the company.

(3) Employees should improve their perception of corporate social responsibility. Considering that the psychological contract has a significant and complete intermediary effect between the external corporate social responsibility and the employee turnover intention, and the psychological contract plays a significant part of the intermediary role between the internal corporate social responsibility and the employee turnover intention, SME employees should continuously improve their perception of corporate social responsibility, so as to form different psychological contracts and then make correct behavioral decisions. Employees should improve their perception of corporate social responsibility. On the one hand, employees can keep abreast of the evaluation information of the company's fulfillment of social responsibilities by continuously expanding the contact channels with the external public or social media, so as to judge whether the company has a good social reputation and image. Based on this, employees can better perceive the fulfillment of corporate social responsibilities, and then influence their attitudes or behavioral decisions. On the other hand, employees can fully understand or perceive the fulfillment of social responsibilities within the company by strengthening the interactive learning and knowledge sharing with other members, and use this as a basis for judging whether to leave or make behavioral decisions.

## Research Insufficiency and Future Prospects

Although this paper has enriched the research scope of social identity theory and social exchange theory to a certain extent, and provided useful reference for SMEs to effectively fulfill their social responsibilities and reduce employee turnover intention, it still has limitations. On the one hand, this paper separately explored the impact of internal and external corporate social responsibilities on employee turnover intention. It did not analyze or test whether there was a synergistic effect between internal and external corporate social responsibilities, and clarified whether the two were complementary or substitutes for each other. Follow-up research can further explore the interaction between internal and external social responsibilities and its effects. On the other hand, this paper explored the intermediate conduction effect of psychological contract in the process of SMEs social responsibility influencing employee turnover intention, and did not consider the possible role of other intermediary variables or contingency factors on this influencing process. Follow-up research can further broaden the research boundary, and fully consider the contingency effects of other contextual variables on the relationship between the social responsibility of SMEs and the employee turnover intention.

## Data Availability Statement

The raw data supporting the conclusions of this article will be made available by the authors, without undue reservation.

## Author Contributions

The content writing and data processing of this article have been completed by ZH.

## Funding

The research was supported by the Taishan Scholars Project Special Fund of Shandong Province of China (tsqn202103095).

## Conflict of Interest

The author declares that the research was conducted in the absence of any commercial or financial relationships that could be construed as a potential conflict of interest.

## Publisher's Note

All claims expressed in this article are solely those of the authors and do not necessarily represent those of their affiliated organizations, or those of the publisher, the editors and the reviewers. Any product that may be evaluated in this article, or claim that may be made by its manufacturer, is not guaranteed or endorsed by the publisher.
